# Frequency and Indications for Anesthesia Exposure in a Pediatric Traumatic Brain Injury Cohort

**DOI:** 10.7759/cureus.88093

**Published:** 2025-07-16

**Authors:** Irim Salik, Sima Vazquez, Iwan Sofjan, Richard Wang, Eris Spirollari, Jared Cooper, Gabriela Rodriguez, Dylan Stewart, Alexander Mittnacht

**Affiliations:** 1 Department of Anesthesiology, Westchester Medical Center, Valhalla, USA; 2 Department of Anesthesiology, New York Medical College, Valhalla, USA; 3 Department of Neurological Surgery, New York Medical College, Valhalla, USA; 4 Department of Neurological Surgery, Westchester Medical Center, Valhalla, USA; 5 Department of Pediatric Surgery, Westchester Medical Center, Valhalla, USA

**Keywords:** anesthesia exposure, morbidity and mortality, outcomes, pediatric traumatic brain injury, traumatic brain injury

## Abstract

Background: Pediatric traumatic brain injury (pTBI) is a leading cause of morbidity in the pediatric population. These patients often undergo multiple anesthetic exposures (AE) during hospitalization for their injuries. In this retrospective study, we sought to analyze the hospital course and outcomes in pTBI patients, hypothesizing that these patients required multiple AEs and that repeated AEs increased the risk for morbidity.

Methods: A chart review of pTBI patients from the Westchester Medical Center Pediatric Trauma Registry was conducted from 2012 to 2021. Demographics, injury data, and hospital course were extracted. Patients were grouped based on the number of AEs, and the hospital course was compared between the groups. Outcomes studied included indications for AE, infection rate, discharge disposition, and new morbidity.

Results: Three hundred and five patients were admitted within the studied period. Increasing AE was associated with worse Injury Severity Scores (ISS) and lower Glasgow Coma Scale (GCS) scores (p < 0.001), as well as with higher rates of intubation, intensive care unit (ICU) admission, external ventricular drain placement, intracranial pressure (ICP) monitor placement, seizure rates, prolonged hospital length of stay (LOS), and decreased likelihood to be discharged home (p < 0.001). On multivariate analysis, AE was associated with infection (p < 0.001) and new morbidity (p = 0.020).

Conclusions: In a cohort of pTBI patients, our review confirmed high anesthesia resource utilization and an increased risk of hospital-acquired infections and new morbidity in patients undergoing multiple anesthetic exposures. Future controlled trials are needed to evaluate the effects and better understand the risk-benefit ratio of multiple procedures and AEs in pTBI patients.

## Introduction

Traumatic brain injury (TBI) is a leading cause of morbidity and mortality in the pediatric population [1]. Our institution is a Level I trauma center with a dedicated pediatric hospital, serving a large geographic referral area. To better understand the complicated hospital course and outcomes in this vulnerable patient population, we created an institutional pediatric traumatic brain injury (pTBI) registry. In this single-center retrospective study, we queried our institutional TBI database to assess the procedural and anesthetic needs of patients with pTBI during their initial hospital stay. We hypothesized that patients with pTBI are resource-intensive in that they require multiple anesthetic exposures (AE) to facilitate their care. Our primary aim was to assess the number and type of AEs required to facilitate therapeutic and diagnostic procedures in pTBI patients during their initial hospital admission. This information is essential as it allows adequate and efficient healthcare resource planning. A secondary hypothesis was that multiple procedures and repeated AEs may increase the risk for newly acquired morbidity, defined in this context as new clinical diagnoses such as infection or respiratory complications resulting from procedural exposure. Consequently, we explored a possible association between AE and hospital-acquired infection, new-onset seizures, intensive care unit (ICU), hospital length of stay (LOS), and discharge disposition.

## Materials and methods

Data and patient cohort

A retrospective chart review was conducted involving pediatric patients under the age of 14 who sustained TBI. The study period spanned from July 12, 2012, to July 23, 2021, and included patients identified from the Westchester Medical Center Pediatric Trauma Registry. Inclusion criteria were based on diagnosis codes corresponding to TBI, specifically utilizing International Classification of Diseases, Ninth Revision (ICD-9) and ICD-10 codes to ensure accurate case identification [2-3]. Pediatric patients were defined according to the institutional criteria, which designate the upper age limit for pediatric neurosurgical and trauma care as 14 years. Consequently, adolescents aged 14 and older were excluded from the cohort, as they are managed by adult trauma and neurosurgical services at the institution.

To collect detailed data, an individual chart review was conducted for each patient by trained research personnel. These reviewers abstracted data on the number, timing, and types of AEs, with attention paid to the specific indications for each anesthetic administration. AE was defined as any administration of general, regional, or procedural anesthesia requiring monitored anesthesia care or sedation during hospitalization.

This study received approval from the Institutional Review Board at Westchester Medical Center, Valhalla, NY (IRB #14779), which also granted a waiver of informed consent due to the retrospective nature of the analysis.

Patient characteristics and outcomes studied

For each patient, demographic information (age, sex, and race/ethnicity) was extracted from electronic medical records. The Area Deprivation Index (ADI) is a measure of the social and environmental factors that impact human biology and was used to assess our cohort further [4]. Clinical variables included the mechanism of injury, Glasgow Coma Scale (GCS) score upon presentation, and whether the patient required intensive care or operative intervention. Additional data collected included imaging findings, Injury Severity Score (ISS), and need for mechanical ventilation.

Patients were stratified into three groups based on the number of anesthesia exposures: no exposure, single exposure, and multiple exposures (>1). Comparisons were made across these groups to evaluate differences in clinical trajectory and outcomes.

Primary outcomes included the frequency, indication, and association of AE with pTBI severity and hospital course. Secondary outcomes included in-hospital infection rates (e.g., pneumonia, urinary tract infection, surgical site infection), length of stay in the hospital and ICU, discharge disposition (home, rehabilitation facility, skilled nursing facility), and new morbidity at the time of discharge, assessed via clinical documentation and follow-up notes.

Statistical analysis

Anesthesia exposure was treated as a continuous variable in regression models to assess its relationship with outcomes. The Kolmogorov-Smirnov test was applied to assess whether continuous variables were normally distributed. Normally distributed variables were analyzed using the Student’s t-test, while Mann-Whitney U tests were used for non-normally distributed variables. Categorical variables were compared using chi-square or Fisher’s exact test, as appropriate.

Multivariate logistic regression models were constructed to evaluate the association between anesthesia exposure and secondary outcomes, adjusting for potential confounders such as age, sex, injury severity, and initial GCS. The level of statistical significance was set at p < 0.05.

All analyses were performed using IBM SPSS Statistics for Windows, Version 28.0 (IBM Corp., Armonk, NY, released 2020).

## Results

Baseline characteristics

There were 305 patients admitted to our institution with TBI between 07/12/2012 and 07/23/2021. The average age was 5.41 ± 5.18 years, and 39.0% (N = 119) of the cohort consisted of females. Most patients were admitted with skull fractures (76.7%, N = 234). Other TBIs represented include subdural hematomas (27.2%, N = 83), intraparenchymal bleeds (24.9%, N = 76), subarachnoid hemorrhages (21.0%, N = 64), and epidural hematomas (10.5%, N = 32). 23.9% (N = 73) of patients had other injuries at the time of TBI admission. There were no associations between exposure to anesthesia, demographic variables, and area deprivation index (Table 1). Increasing AE was associated with worse ISS scores (p < 0.001) and lower GCS scores (p < 0.001). Patients with higher AE requirements were associated with higher rates of epidural (p < 0.001), subarachnoid bleed (p < 0.001), intraparenchymal bleed (p < 0.001), or other non-cranial injuries (p < 0.001) (Table 1).

**Table 1:** Demographics and injury characteristics of patients with no AE, 1 AE or more than 1 AE Cells show descriptive (N, %) statistics unless otherwise noted. P-value is based on linear regression with AE as a continuous variable. Significance is considered at P-values <0.05 AE: anesthesia exposure; AE 0: no anesthesia exposure; AE 1: one anesthesia exposure; AE > 1: more than one anesthesia exposure

Demographics and injury characteristics	Total (N=305)	AE 0 N=192 (63.0%)	AE 1 N= 75 (24.6%)	AE > 1 N=38 (12.5%)	P-value
Demographics					
Age, mean ± stdv	5.41 ± 5.18	5.09 ± 5.10	5.82 ± 5.18	6.20 ± 5.61	0.018
Female gender	119 (39.0%)	78 (40.6%)	28 (37.3%)	13 (34.2%)	0.935
Black race	15 (4.9%)	11 (5.7%)	3 (4.0%)	1 (2.6%)	0.476
Caucasian race	204 (66.9%)	128 (66.7%)	49 (65.3%)	27 (71.1%)	0.909
Hispanic race	63 (20.7%)	36 (18.8%)	18 (24.0%)	9 (23.7%)	0.170
Area Deprivation Index, mean ± stdv	27.80 ± 21.64	26.99 ± 21.68	29.95 ± 21.71	27.66 ± 21.59	0.981
Medicaid Insurance	146 (47.9%)	85 (44.3%)	42 (56.0%)	19 (50.0%)	0.159
Injury characteristics					
Injury Severity Score, mean ± stdv	13.40 ± 9.95	10.46 ± 7.6	15.41 ± 9.38	24.26 ± 12.61	< .001
Glasgow Coma Scale, mean ± stdv	13.44 ± 3.65	14.44 ± 2.16	12.80 ± 4.23	9.63 ± 5.38	< .001
Skull fracture	234 (76.7%)	150 (78.1%)	57 (76.0%)	27 (71.1%)	0.618
Epidural hemorrhage	32 (10.5%)	8 (4.2%)	11 (14.7%)	13 (34.2%)	< .001
Subdural hemorrhage	83 (27.2%)	46 (24.0%)	23 (30.7%)	14 (36.8%)	0.501
Subarachnoid hemorrhage	64 (21.0%)	30 (15.6%)	23 (30.7%)	11 (28.9%)	< .001
Intraparenchymal hemorrhage	76 (24.9%)	38 (19.8%)	23 (30.7%)	15 (39.5%)	< .001
Non-cranial injuries sustained	73 (23.9%)	29 (15.1%)	26 (34.7%)	18 (47.4%)	< .001

AE

**Figure 1:**
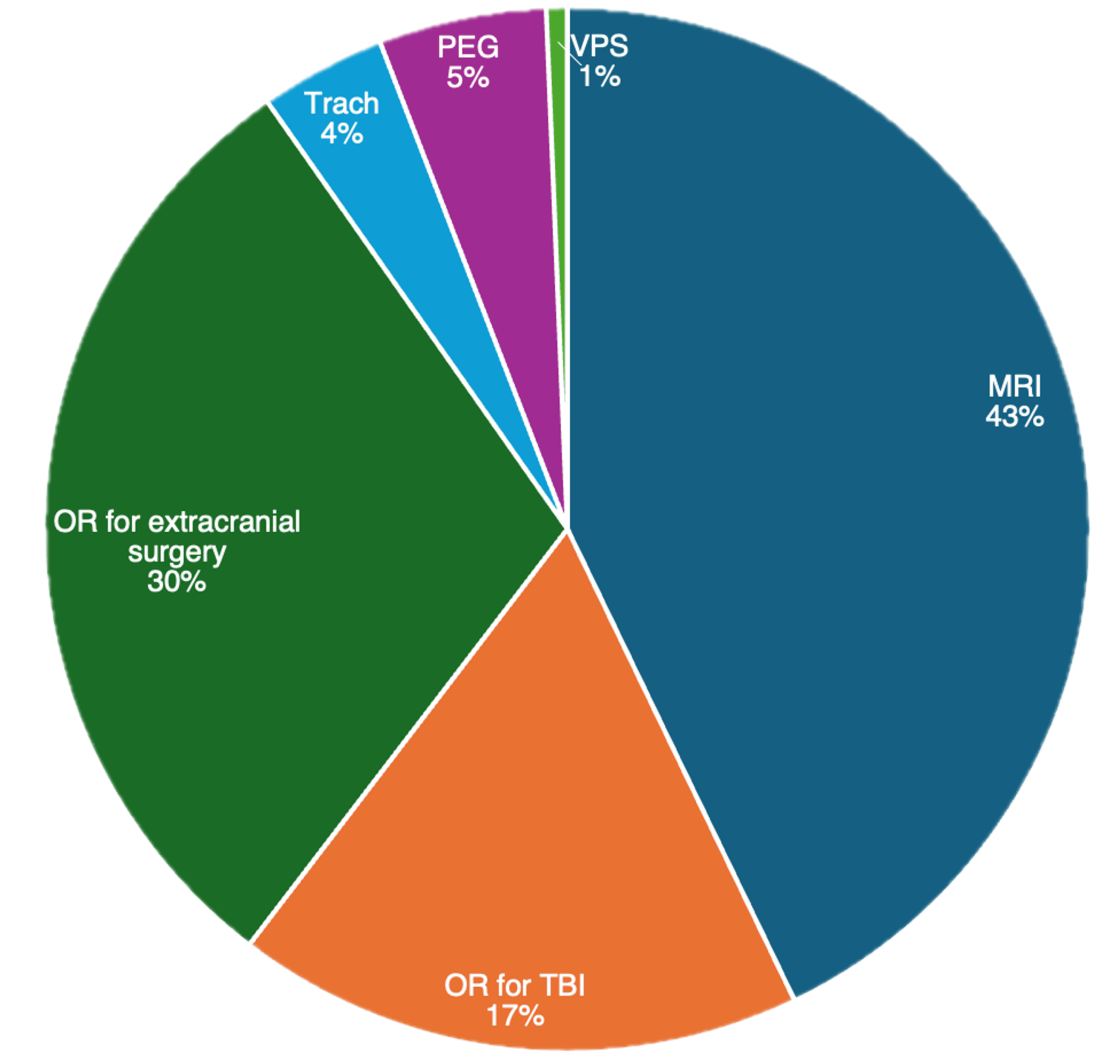
Pie chart showing the breakdown of indications for AE in our cohort AE: anesthesia exposure; VPS: ventriculoperitoneal peritoneal shunt; MRI: magnetic resonance imaging; Trach: tracheotomy; OR: operating room; TBI: traumatic brain injury; PEG: percutaneous endoscopic gastrotomy

**Figure 2:**
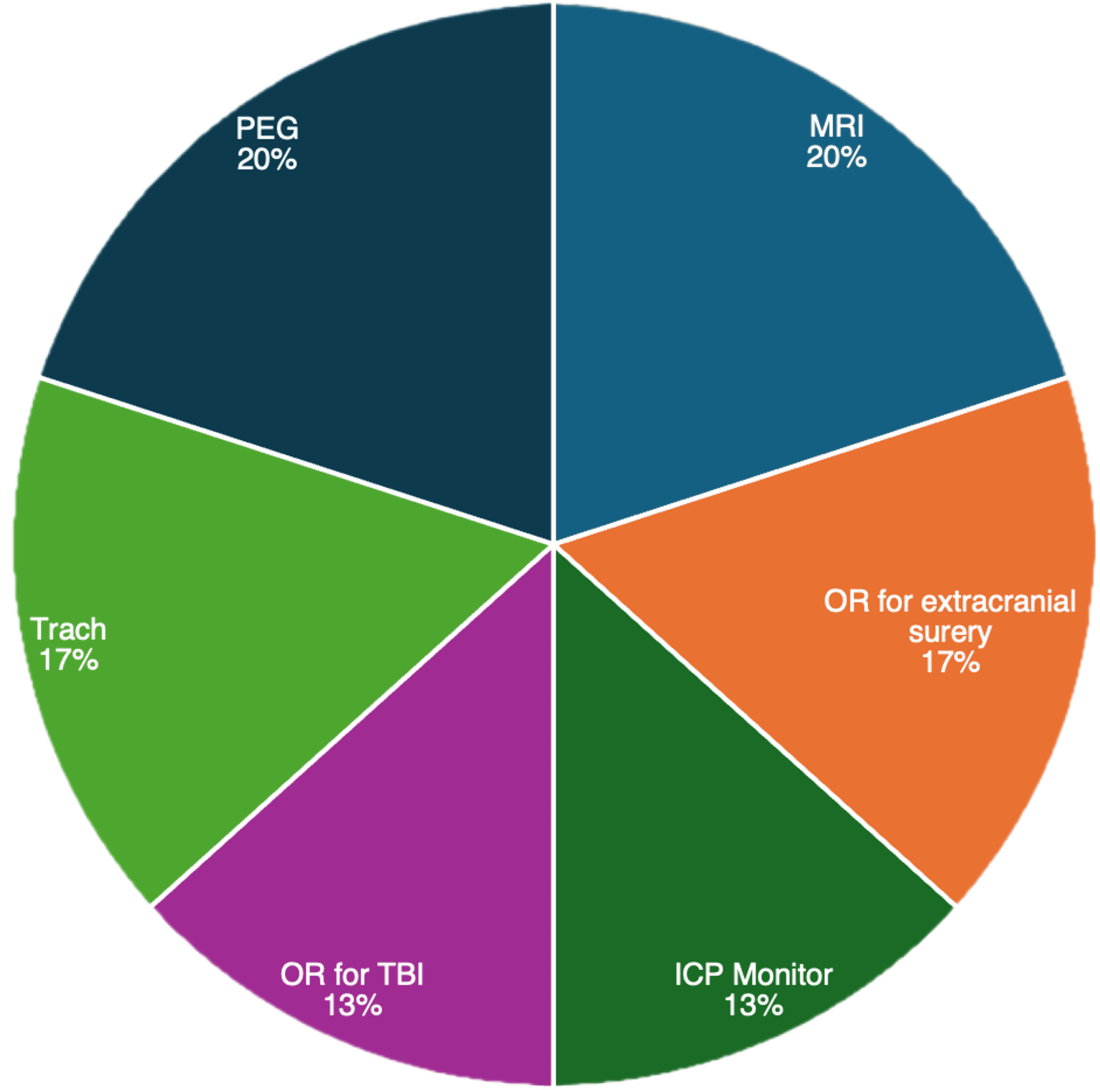
Pie chart showing the breakdown for AEs in patients who developed infection AE: anesthesia exposure; MRI: magnetic resonance imaging; OR: operating room; ICP: intracranial pressure; Trach: tracheotomy; PEG: percutaneous endoscopic gastrotomy

The main indications for AE include MRI (42.9%, N = 64), extracranial surgery (29.9%, N = 45), and surgery for TBI (17.5%). Percutaneous endoscopic gastrostomy (PEG) tube placement (5.2%), tracheotomy (3.9%), and ventriculoperitoneal shunt (VPS) placement (0.6%) were the other indications for AE (Figure 1). Of patients who had an MRI with anesthesia, 51 (77.3%) underwent one MRI with anesthesia, and 13 (19.7%) underwent two MRIs with anesthesia. Of patients who underwent extracranial surgery, 38 (82.6%) patients underwent one extracranial surgery, four (8.7%) underwent two surgeries, and three (6.5%) underwent three surgeries. Of all patients, 113 (37.1%) had one or more AEs during their pTBI hospitalization. There were no differences in age, sex, ethnicity/race, or mean ADI between those with one or fewer AE and those with more than one AE (Table 1). Twelve of 17 (70.5%) patients with more than one AE received an MRI with anesthesia (Figure 2). An increasing number of AEs was associated with higher rates of intubation, ICU admission, ICU LOS, external ventricular drain (EVD) placement, intracranial pressure (ICP) monitor placement, seizure, infection, and longer LOS (p < 0.001 for all) (Table 2). Most (87.9%, N = 268) of patients were discharged home. Patients with increasing AE were less likely to be discharged home (p < 0.001). 4.3% (N = 8) of patients were discharged with a new morbidity, and 3.0% (N = 9) died within one year of admission. AE was associated with an increased rate of discharge with new morbidity within each setting (Table 3). Associations between the number of AEs and measured outcomes are presented in Table 2.

**Table 2:** Multivariate logistic regressions assessing factors associated with outcomes of infection, discharge home, new morbidity, and mortality Significance is considered at P-values <0.05. OR: odds ratio; AE: anesthesia exposure; LOS: length of stay

Outcomes measures	Infection		Discharge home		New morbidity		Mortality	
	OR (95%CI)	P-value	OR (95%CI)	P-value	OR (95%CI)	P-value	OR (95%CI)	P-value
Age	0.89 (0.69-1.14)	0.360	0.87 (0.77-0.99)	0.031	0.93 (0.80-1.08)	0.357	1.07 (0.82-1.38)	0.634
Female	2.64 (0.32-21.78)	0.366	1.84 (0.45-7.54)	0.396	0.84 (0.20-3.53)	0.812	0.10 (0.00-3.90)	0.220
Injury Severity Score	1.07 (0.99-1.17)	0.108	0.90 (0.84-0.96)	< .001	1.01 (0.95-1.08)	0.692	1.11 (0.95-1.30)	0.186
Glasgow Coma Scale	0.82 (0.63-1.06)	0.124	1.32 (1.17-1.50)	< .001	0.81 (0.69-0.95)	< .001	0.66 (0.48-0.91)	0.012
LOS	1.00 (0.97-1.04)	0.841	0.98 (0.95-1.00)	0.052	1.01 (0.99-1.03)	0.634	0.57 (0.35-0.90)	0.016
AE	2.60 (1.27-5.33)	< .001	0.60 (0.31-1.18)	0.136	1.66 (1.08-2.55)	0.020	4.71 (0.56-39.55)	0.154

**Table 3:** Hospital course and discharge disposition in patients with no AE, 1 AE or more than 1 AE. Cells show descriptive (N, %) statistics unless otherwise noted. All continuous variables are presented as mean±standard deviation (SD). P-value is based on linear regression with AE as a continuous variable. Significance is considered at P-values <0.05. AE: anesthesia exposure; AE 0: no anesthesia exposure; AE 1: one anesthesia exposure; AE > 1: more than one anesthesia exposure; ED: emergency department; ICU: intensive care unit; LOS: length of stay; ICP: intracranial pressure; EVD: external ventricular drain

Hospital course and discharge disposition	Total N=305	AE 0 N=192 (63.0% )	AE 1 N=75 (24.6%)	AE > 1 N=38 (12.5%)	P-value
Hospital course					
Intubated	60 (19.7%)	9 (4.7%)	24 (32.0%)	27 (71.1%)	< .001
On scene	27 (8.9%)	6 (3.1%)	11 (14.7%)	10 (26.3%)	< .001
In the ED	27 (8.9%)	3 (1.6%)	9 (12.0%)	15 (39.5%)	< .001
ICU	190 (62.3%)	104 (54.2%)	54 (72.0%)	32 (84.2%)	< .001
ICU LOS	2.73 ± 5.78	1.34 ± 3.46	2.53 ± 2.98	9.97 ± 11.37	< .001
EVD	15 (4.9%)	2 (1.0%)	3 (4.05%)	10 (26.3%)	< .001
ICP monitor	9 (3.0%)	1 (0.5%)	2 (2.7%)	6 (15.8%)	< .001
Seizure during admission	22 (7.2%)	6 (3.1%)	11 (14.7%)	5 (13.2%)	< .001
Infection	9 (3.0%)	0 (0%)	1 (1.3%)	8 (21.1%)	< .001
LOS	6.22 ± 23.47	4.31 ± 26.58	5.44 ± 5.04	17.42 ± 26.10	< .001
Discharge disposition					
Discharge home	268 (87.9%)	187 (97.4%)	61 (81.%)	20 (52.6%)	< .001
Discharged with new morbidity	13 (4.3%)	2 (1.0%)	3 (4.0%)	8 (21.1%)	< .001
Died in hospital or within 1 year	9 (3.0%)	2 (1.0%)	5 (6.7%)	2 (5.3%)	0.172

 Independent association between AE and outcomes 

On multivariate analysis, AE was associated with infection (p < 0.001) and new morbidity (p = 0.020) (Table 3). The most common indications for AE in patients who developed infection were MRI (66.7%, N = 6) and PEG placement (66.7%, N = 6). This was followed by extracranial surgery (55.6%, N = 5), tracheostomy (55.7%, N = 5), and surgery for TBI (44.4%, N = 4). Three (33.3%, N = 3) patients who developed infection had more than four AEs. Details of AE for patients who developed infections are outlined in Table 4.

**Table 4:** Indications for AE in patients with infection AE: anesthesia exposure; OR: operating room; MRI: magnetic resonance imaging; PEG: percutaneous endoscopic gastrotomy; Trach: tracheotomy Note: The numerical identifiers (e.g., 1, 2, 3, etc.) used in the table are arbitrary and were created solely for the purpose of referencing specific cases within this article. These identifiers do not correspond to any patient-identifying information.

Patient	AE 1	AE 2	AE 3	AE 4	AE 5	AE 6	AE 7	AE 8
1	OR for TBI	Extracranial surgery						
2	MRI	MRI						
3	MRI	MRI	MRI	PEG				
4	MRI	MRI	Trach	PEG	Extracranial surgery	Extracranial surgery	Extracranial surgery	Extracranial surgery
5	MRI	MRI	OR for TBI	Trach	PEG	Extracranial surgery	Extracranial surgery	Extracranial surgery
6	Surgery for TBI	Trach	PEG					
7	MRI	MRI	OR for TBI	Trach	PEG	Extracranial surgery	Extracranial surgery	Extracranial surgery
8	OR for TBI	Trach	PEG	Extracranial surgery	Extracranial surgery			
9	MRI							

## Discussion

Our retrospective review of pTBI patients found that injury is resource-intensive, with 37% (N = 113) of patients requiring one or more AEs for facilitation of diagnostic and therapeutic procedures. On further analysis, AE was independently associated with hospital-acquired systemic infection, irrespective of ISS or hospital LOS. Although our sample size was small, more than ¾ of our cohort who developed infection required anesthesia for surgical intervention, including extracranial surgery in up to 30% (N = 3) of patients, ICP monitor placement, intracranial surgery directly associated with their TBI, gastrostomy tube placement, and tracheostomy.

Adverse functional outcomes and impaired executive function have previously been reported by Roberts and colleagues in adult patients undergoing extracranial surgery following TBI, providing further evidence of AE as a potential risk factor for poor outcomes in TBI patients. This group recommends screening patients with recent TBI and avoiding elective extracranial procedures unrelated to their TBI for several weeks, as changes in blood-brain barrier permeability, edema, and physiologic perturbations such as hypotension and hypoxemia can adversely impact the acute TBI population [5]. Similarly, Abcejo and colleagues retrospectively reviewed the utilization of anesthesia for patients following mild TBI, identifying the greatest need for anesthetic care for surgical and diagnostic procedures within one week and one month of injury, respectively, to facilitate procedures considered elective or unrelated to the original concussive injury. Mild TBI patients had higher visual analog pain scores and increased postoperative headaches following AE [6]. The authors recommend increased awareness of the neurophysiological changes in the acutely concussed patient to avoid secondary cognitive insults.

The most common indication for AE in our pTBI cohort was for diagnostic imaging procedures, namely, MRI. To facilitate MRI, almost all pTBI patients receive general anesthesia under the direct or indirect care of an attending physician or an anesthesiologist at our institution. For patients who had more than one AE, most patients had either a single or repeat MRI brain scan following TBI, with a minority receiving greater than two scans. There are concerns regarding the overuse of MRI in the trauma setting [7]. Computed tomography (CT) scans without anesthesia remain the first-line imaging for most pTBI patients, although large cohort studies have shown an increased risk of malignancy from ionizing radiation in children [8-11]. The Pediatric Emergency Care Applied Research Network (PECARN) has developed criteria for clinical decision-making in trauma patients with low risk for TBI who may be able to forego imaging. Despite these recommendations, the use of CT scans in the setting of mild TBI has not declined [10,12,13]. At our institution, the PECARN criteria are utilized prior to undergoing radiologic imaging, which is primarily with CT. An MRI with anesthesia is undertaken within 48 hours to evaluate TBI sequelae in hospitalized patients. Alternatively, rapid MRI without anesthesia has been touted as an excellent alternative modality to CT to evaluate pTBI, with numerous studies showing adequate sensitivity and specificity of rapid MRI in detecting TBI sequelae in non-sedated toddlers [14, 15]. However, MRI remains superior to CT in the diagnosis of intraparenchymal lesions, with a higher detection rate for subdural hemorrhage, subarachnoid hemorrhage, and epidural hematoma [16].

In our study, patients with more AEs were those with more severe TBI, including a higher mean ISS and an increased risk of multiple injuries. These patients also had higher odds of mechanical ventilation and ICU admission, along with an increased risk of intensive procedures such as EVD and ICP monitor placement. More severe TBI patients were also less likely to be discharged home and more likely to develop new morbidity as a function of their underlying TBI. Although these results may not be surprising, interestingly, patients with multiple AEs did not experience a higher mortality rate. It is apparent that patients with worse injuries would require a higher level of care, more imaging requirements as a follow-up to their underlying injury, and more neurosurgical interventions. While the utilization of anesthesia for these interventions is often unavoidable, our study findings add to the existing data that more elective anesthetics and procedures should possibly be avoided in pTBI patients due to the risk of secondary cerebral insults.

The most pertinent finding of our study was an increased risk of hospital infection associated with multiple AEs. Although an exact causal relation would require a much larger patient cohort, there is no doubt that postoperative infections are associated with a significant increase in morbidity and mortality [17]. Several anesthesia-related factors may play a role in infection control, including safe injection practices, hand hygiene, equipment reprocessing issues, and environmental contamination [18-22]. The most common indications for AE in our patients who developed a hospital-acquired infection were MRI imaging and PEG tube placement, followed by extracranial surgery and tracheostomy; two-thirds of patients who acquired infection had suffered a skull fracture. Early tracheostomy and gastrostomy tube placement are often performed simultaneously to prevent complications of prolonged endotracheal intubation, shorten hospital length of stay, and prevent malnutrition following TBI [23]. Nabika and colleagues found an increased risk of VPS infection with concomitant PEG tube placement, suggesting that waiting a month between procedures may be beneficial, particularly if the shunt is placed after the gastrostomy tube [24].

Our study is limited by its retrospective nature. We found an increased infection risk in patients with multiple AEs. Although we attempt to utilize multivariate regression analysis, there remains a risk of unmeasured confounding with retrospective data, particularly regarding increased hospital infection risk in our pTBI cohort. Granular variables, including lab values, immunity status, and detailed imaging, were not available for analysis in our dataset. This study evaluated in-hospital complications but not long-term complications of pTBI and repeated AE, including neurodevelopmental outcomes and functional status following hospital discharge. While the literature does not provide standardized practice guidelines for the delay of elective anesthetics in TBI patients, the highest utilization of anesthetic care is frequently soon after a TBI, when the brain may be vulnerable to secondary insults. Many would argue that the utilization of anesthesia is not elective in this vulnerable population that requires multiple diagnostic and surgical interventions expeditiously to facilitate care.

## Conclusions

In a cohort of pTBI patients, our retrospective review confirmed high anesthesia resource utilization, with 37% requiring one or more anesthetics, mainly for diagnostic imaging and extracranial surgical procedures. Additionally, we found an increased risk of hospital-acquired infections and new morbidity in patients undergoing multiple procedures under anesthesia. Future prospective controlled trials are needed to evaluate the effects and better understand the risk-benefit ratio of multiple procedures and AEs in pTBI patients.
